# Draft Genome Sequence of *Lactobacillus plantarum* Strain IPLA 88

**DOI:** 10.1128/genomeA.00524-13

**Published:** 2013-07-25

**Authors:** Victor Ladero, Patricia Alvarez-Sieiro, Begoña Redruello, Beatriz del Rio, Daniel M. Linares, M. Cruz Martin, María Fernández, Miguel A. Alvarez

**Affiliations:** Instituto de Productos Lácteos de Asturias, IPLA-CSIC, Villaviciosa, Asturias, Spain

## Abstract

Here, we report a 3.2-Mbp draft assembly for the genome of *Lactobacillus plantarum* IPLA 88. The sequence of this sourdough isolate provides insight into the adaptation of this versatile species to different environments.

## GENOME ANNOUNCEMENT

*Lactobacillus plantarum* shows great capacity to adapt to different environments, a consequence of its versatile metabolism. It is encountered in several habitats, including human mucosae and fermented foods and beverages (especially those derived from vegetables) (1–3). Some *L. plantarum* strains have been proposed as probiotics (4–6) or as agents for eliminating toxic compounds, such as biogenic amines (7). Over the past decade, several groups have focused on the role of *L. plantarum* in sourdough fermentation and suggest it to be an ideal starter for type I sourdoughs (8). We report here the draft genome of *L. plantarum* IPLA 88 strain, isolated from an Italian type I sourdough.

Total DNA from *L. plantarum* strain IPLA 88 was extracted, and a genomic library of 0.5 kbp was constructed and subjected to 90-bp paired-end sequencing (providing approximately 120-fold coverage) using an HiSeq 1000 system sequencer (Illumina) at the Beijing Genomics Institute (China). Quality-filtered reads were assembled using Velvet software (http://www.ebi.ac.uk/~zerbino/velvet/), resulting in 208 contigs ranging from 208 to 205,844 bp. The total sequence length is 3,254,055 bp, with a G+C content of 44.4%. The latter is consistent (at the low end of the range) with results reported for other *L. plantarum* genomes (44.5 to 44.7%). Annotation was performed using the NCBI Prokaryotic Genomes Annotation Pipeline (PGAAP) (http://www.ncbi.nlm.nih.gov/genomes/static/Pipeline.html) and improved with results obtained by BLAST analysis (http://blast.ncbi.nlm.nih.gov). The genome contains 3,116 predicted coding sequences. Predicted copies of the 16S, 23S and 5S rRNA genes were found, as well as 47 genes for tRNAs.

The strain possesses genes coding for three alpha-amylases (L103_0182, L103_09189, and L103_13633) and one oligo-1,6-glucosidase (L103_09184) (providing the ability to hydrolyze α-1,6-d-glucosidic linkages in starch and glycogen) and for several peptidases specific for glutamine and proline residues (very abundant in wheat gluten proteins); these help IPLA 88 grow and participate in sourdough fermentation (9). No evidence of virulence-related or antibiotic resistance genes was found, and no genes involved in biogenic amine production were detected. The genome did, however, contain at least three prophages plus some genes involved in plasmid mobilization or replication, and one putative toxin/antitoxin plasmid stabilization system, in agreement with the presence of at least three plasmids (data not shown).

In comparisons with the genomes of other *L. plantarum* strains in public databases, including *L. plantarum* WCFS1 (4), JDM1 (5), and ZJ316 (6) of human origin and *L. plantarum* ST-III (10) of dairy origin, the *L. plantarum* IPLA 88 genome showed 230 to 419 different genes, of which 157 were unique to the strain. This may be a reflection of its different origin. Although 65% of these unique genes code for proteins of unknown function, some appear to be related to exopolysaccharide synthesis or to encode peptidase activities, capacities that are desirable for the development of sourdough texture and flavor.

Knowledge of the *L. plantarum* IPLA 88 genome sequence should help improve our understanding of how lactic acid bacteria in general, and *L. plantarum* in particular, adapt to different environments.

### Nucleotide sequence accession numbers.

The results of this whole-genome shotgun project were deposited in the DDBJ/EMBL/GenBank database under the accession no. ASJE00000000. The version of the genome described here has the accession number ASJE01000000.
